# The Hospital World

**Published:** 1910-10-15

**Authors:** 


### THE HOSPITAL WORLD.

### Elections and Appointments.

LADY CLANMORRIS has been appointed President of the Bangor Cottage Hospital, of which Mr. John McMeekan, J.P., is chairman, and Mr. James Milliken and Mr. David Orr are joint hon. secretaries.

THE House Committee at the North Devon Infirmary has been elected as follows: Mr. W. Richards, Mr. C. E. R. Chanter, Mr. A. P. Kent, Mr. G. Norman, Mr. A. E. Barnes, Capt. Campbell, Mr. R. P. Dunn Pattison, the Rev. J. Jarratt, the Rev. A. R. Fuller, the Rev. A. B. S. Wrey, Major H. Chichester, Mr. G. F. Fisher, and Mr. W. Z. Holmes.

### Personalia.

THE KING AND QUEEN have become patrons of the Queen Victoria Memorial Hospital, Nice, and the King has become patron of the Royal National Sanatorium for Consumption and Diseases of the Chest, Bournemouth.

To mark the presentation of *x*-ray apparatus by Sir David Salomons to the General Hospital, Tunbridge Wells, the treasurer and matron have been presented with a sapphire pin and brooch respectively, the stones having been changed to a yellow colour by the emanations of radium.

